# Harnessing Halogenated Zeolitic Imidazolate Frameworks for Alcohol Vapor Adsorption

**DOI:** 10.3390/molecules29245825

**Published:** 2024-12-10

**Authors:** Kevin Dedecker, Martin Drobek, Anne Julbe

**Affiliations:** Institut Européen des Membranes (IEM), CNRS, ENSCM, Univ Montpellier, Place Eugène Bataillon, 34095 Montpellier, France

**Keywords:** zeolitic imidazolate framework, volatile organic compounds, alcohol, adsorption, separation, halogenated

## Abstract

This study explores Zeolitic Imidazolate Frameworks (ZIFs) as promising materials for adsorbing alcohol vapors, one of the main contributors to air quality deterioration and adverse health effects. Indeed, this sub-class of Metal–Organic Frameworks (MOFs) offers a promising alternative to conventional adsorbents like zeolites and activated carbons for air purification. Specifically, this investigation focuses on ZIF-8_Br, a brominated version of ZIF-8_CH_3_, to evaluate its ability to capture aliphatic alcohols at lower partial pressures. The adsorption properties have been investigated using both experimental and computational methods combining Density Functional Theory and Grand Canonical Monte Carlo simulations. The Ideal Adsorbed Solution Theory (IAST) has been used to assess the material selectivity in the presence of binary equimolar alcohol mixtures. Compared to ZIF-8_CH_3_, the brominated analog has been shown to feature a higher affinity for alcohols, a property that could be advantageously exploited in environmental remediation or in the development of membranes for alcohol vapor sensors.

## 1. Introduction

Air quality is currently raising great concerns due to its significant impact on human health. It has been shown that anthropogenic pollution was responsible for 7.5% of all deaths on Earth in 2016 [1]. The emission of Volatile Organic Compounds (VOCs) appears to be one of the main factors contributing to air quality degradation. Population integrity may be affected either by direct exposure to VOCs, leading to respiratory problems [2,3], neurological symptoms [4,5], and cancers [6,7] or indirectly through their conversion into ozone [8], a powerful oxidant that causes severe and persistent respiratory diseases [9].

Among the existing types of VOCs, the alcohol sub-class occupies a particular place due to the deleterious effects accentuated by their omnipresence in industrialized societies. For instance, methanol, n-propanol, and isopropanol, are widely found in household products, cleaning agents, and personal care items. In general, their toxicity depends on both their concentration and exposure duration [10]. In liquid form, methanol notoriously induces blindness when ingested, while its vapors can cause nausea, fatigue, and respiratory irritation [11]. Propanol may cause irritant effects [12], while contact with isopropanol can provoke clinical symptoms of toxicity such as hypoactivity and kidney damages [13]. Collectively, these three alcohols are among the most commonly encountered solvents in daily life (e.g., in commercial cleaning products) that present significant health risks and, therefore, warrant a special attention for their monitoring/elimination.

To protect individuals from these deleterious effects, an appropriate solution relies on a strategic combination of air purification and concentration monitoring using effective adsorbents for the capture and detection of alcohols in the air at low partial pressures. Within the library of adsorbents currently in use (e.g., activated carbons and zeolites), zeolites are often preferred due to their higher hydrophilicity favoring host–guest interactions with polar alcohol molecules [14]. However, their acidic character may lead to catalytic conversion of alcohols into potentially more harmful products [15]. Hence, the search for suitable alcohol sorbents tends to be directed towards other classes of materials that do not present such disadvantage.

Metal–Organic Frameworks (MOFs) [16] represent a class of crystalline hybrid organic-inorganic materials featuring high porosity [17] and rich chemical versatility that enables “nanoscale molecular engineering” such as metal substitution [18,19,20], the functionalization of ligands [21,22,23], and/or the resizing of organic linkers [24,25]. These properties could then be advantageously exploited to adjust their adsorption characteristics for the qualitative and quantitative removal of VOCs [26,27,28,29,30,31,32] and gas storage [33,34].

In the family of MOFs, Zeolitic Imidazolate Frameworks (ZIFs) characterized by zeolite-like topology (e.g., SOD, LTA, and RHO zeolitic structures) represent one of their best-known subclasses [35]. They are generally formed by the association of metal centers with imidazole-type ligands that coordinate through nitrogen atoms located in the aromatic rings.

Over the past decades, ZIFs have attracted significant attention as a potential alternative to zeolites due to their structural versatility [36] and chemical/thermal stability [37]. Moreover, they demonstrate notable framework flexibility [38,39], which is facilitated by the dynamic movement of ligands around the metal–ligand–metal axis, enabling the accommodation of adsorbates larger than the theoretical size of their pore apertures [40,41]. Additionally, it has been shown that their adsorption properties may be fine-tuned through structural engineering. For instance, Economou et al. [42] demonstrated that substituting the metal in the iconic ZIF-8_CH_3_ (zinc 2-methylimidazolate) with a smaller atom (e.g., cobalt) reduced the pore aperture from 3.42 Å to 3.33 Å, which resulted in shorter Co-N bonds. This finding was then exploited to optimize CO_2_/CH_4_ separation performance [43] by precisely adjusting the pore aperture of the mixed Zn/Co-ZIF-8 [44]. Additionally, ligand functionalization has been shown to influence the thermal stability [45], structural flexibility [46] and adsorption properties [47,48,49] of ZIFs. A study by J. Jiang et al. [50] investigated six ZIFs with varied chemical groups for ethanol/water separation, highlighting the crucial role of hydrogen bonding in facilitating interactions with ethanol and water molecules. Therefore, higher adsorption capacities were observed for the hydrophilic ZIFs (e.g., ZIF-90, -96, and -97) with polar groups compared to their hydrophobic counterparts (e.g., ZIF-8, -25, and -71). This conclusion was supported by the investigation of alcohol adsorption in hydrophobic ZIF-8_CH_3_, where alcohols with longer alkyl chains were found to be more easily adsorbed within pores due to their lower polarity [51,52,53].

Although numerous functionalized ZIFs have been thoroughly investigated, it appears that, in general, the reported structures only adsorb alcohols at relatively high partial pressures. However, realistic scenarios generally involve rather lower concentrations of pollutants in the air. Therefore, the search for more performable ZIF structures, suitable for the efficient sorption of pollutants in real conditions, is still ongoing.

In a recent study by Yagi and Ueda [54], ZIF-8_CH_3_ was compared with two of its halogenated analogues, ZIF-8_Cl and ZIF-8_Br, to assess their effectiveness in benzene adsorption. They observed that ZIF-8_CH_3_ demonstrated superior performance, attributed to the presence of CH/π interactions between the imidazolate moiety, including the methyl group, and the π electrons of benzene molecules. Conversely, ZIF-8_Cl and particularly ZIF-8_Br exhibited significantly weaker interactions with benzene. Using spectroscopic analysis, the studied ZIFs were classified based on the strength of their host–guest interactions as follows: ZIF-8_Br << ZIF-8_Cl < ZIF-8_CH_3_. This investigation shows the increased efficiency of ZIF-8_CH_3_ in interacting with π–electron systems and suggests that halogenated analogs, especially ZIF-8_Br, may offer improved performance for the adsorption of non-aromatic (aliphatic) VOCs.

Considering the aforementioned findings, ZIF-8_Br shows great promise for the efficient adsorption of aliphatic alcohols at lower partial pressures, especially compared to the well-known ZIF-8_CH_3_. Hence, driven by this objective, this study seeks to explore the adsorption properties of ZIF-8_Br through both experimental and theoretical methods, aiming to clarify host–guest interactions using a computational approach that integrates DFT and GCMC calculations. The adsorption performance of ZIF-8_Br and ZIF-8_CH_3_ will be compared for the capture of selected primary and secondary alcohols encountered in daily life, presenting a potential health risk in the long term. Furthermore, the obtained findings are expected to contribute to the development of sensors integrating MOFs as membrane layers [55,56,57,58], thus providing effective solutions for monitoring alcohol emissions, reducing individual exposure and mitigating alcohol-related health risks.

## 2. Results and Discussion

### 2.1. Description of MOF Structures

In order to deeply explore the adsorption properties of ZIF derivatives, a first study was conducted, focusing on the assessment of their structural attributes using the Zeo++ software version 0.3. The following results aim to provide new data missing in the current literature, thus offering insights into the characteristics of both ZIF-8_CH_3_ and ZIF-8_Br.

**Figure 1:**
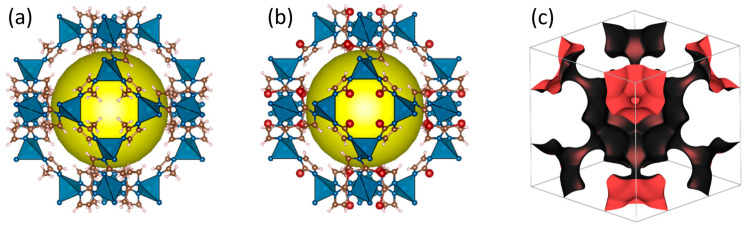
Schematic representation of (**a**) ZIF-8_CH_3_ structure, (**b**) ZIF-8_Br structure, (**c**) network of accessible pores within the ZIF structure, highlighting entry points and interconnected cage cavities.

From a topological point of view, ZIF-8_CH_3_ and ZIF-8_Br are isostructural and have both a cubic structure (space group: I -4 3 m) [54]. They are formed of zinc cations as metal centers coordinated tetrahedrally to either 2-methylimidazolate or 2-bromoimidazolate. The lengths of the resulting Zn-N bonds were determined to be 1.98 and 1.99 Å for ZIF-8_CH_3_ and ZIF-8_Br, respectively. The crystallographic data show the presence of four-membered ring (4MR) and six-membered ring (6MR) windows forming zeolitic cages (Figure 1a,b). Due to their very small sizes, 4MR entries cannot pass any adsorbate. Hence, only the size of the 6MR entries dictates the accommodation of species in the ZIF cavities and confers eight entry points to each cage (Figure 1c). A theoretical analysis of the structural features obtained with Zeo++ reveals some differences between the two structures in terms of pore limiting diameters (PLD), largest cavity diameters (LCD), surface area (SA), and pore volume (V_p_) (Table 1) due to ligand functionalization. The substitution of the -CH_3_ group by -Br induces an increase in the length of the Zn-N bond leading to an expansion of the unit cell volume and a slight shrinkage of the 6MR windows (PLD). In addition, ZIF-8_Br exhibits a lower specific surface area (m^2^/g) and pore volume (cm^3^/g) than its methylated analog due to a significant difference in molar mass between the two materials (2730.86 and 4287.73 g/mol for ZIF-8_CH_3_ and ZIF-8_Br, respectively) and the steric hindrance of the chemical group grafted onto the ligand.

### 2.2. Synthesis and Characterization of MOFs

Particular effort was devoted to the optimization of the ZIF-8_Br synthesis process, in order to shorten its duration and lower the reaction temperature and thus facilitate the overall preparation procedure. Indeed, the ZIF-8_Br synthesis protocol reported in the literature is time-consuming with a heating step of the reaction mixture in an autoclave at 100 °C for 60 h [54].

The synthesis strategy proposed in this work was inspired by a study of Fischer’s group [59] in which the formation of ZIF structures was successfully achieved at room temperature within a short reaction time (~1 h). Compared to the original synthesis protocol [54], zinc nitrate was replaced by zinc acetate, and the solvent (ethanol) was replaced by a DMF/MeOH mixture (1:1). Unlike the application of zinc nitrate in ethanol, the reaction carried out with zinc acetate in DMF/MeOH at room temperature yielded MOF formation already after 1 h. The reason for such a difference in reactivity can be explained by faster and facilitated deprotonation of the ligand triggered by the weak acetate base in solution. Indeed, this phenomenon allows additional coordination of the ligand with the zinc cations. The mechanism relies on the formation of ZIF-8_CH_3_, which involves three successive steps [60]: (i) coordination of 2-methylimidazole to Zn^2+^, (ii) deprotonation of the ligand, and (iii) oligomerization by linking different Zn^2+^ centers via deprotonated 2-methylimidazole ligands. In this context, the kinetics of ZIF-8_Br formation is improved in the presence of acetate anions which represent better proton acceptors than nitrate ions. A similar effect of the metal precursor on crystallization kinetics was also reported by Tu et al. [59] for an isostructural analogue, ZIF-65 (zinc 2-nitroimidazolate).

ZIF-8_CH_3_ was synthesized using zinc nitrate according to the protocol reported in the literature [61]. The formation of this MOF occurs within one hour at room temperature leading to a gradual increase in the turbidity of the reaction solution. The faster kinetics indicate that acetate ions are not essential to obtain ZIF-8_CH_3_ in a short time.

**Figure 2:**
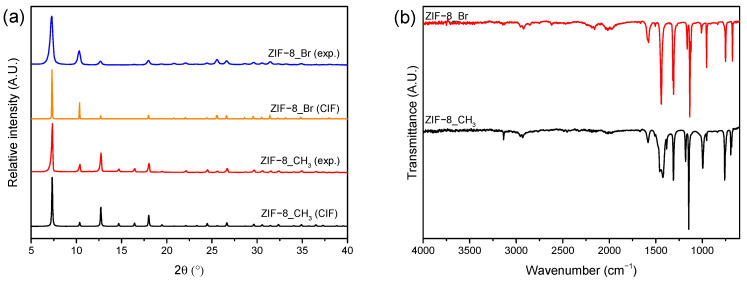
(**a**) Comparison of experimental and simulated PXRD patterns for ZIF-8_CH_3_ and ZIF-8_Br; (**b**) Comparison of FTIR experimental spectra for ZIF-8_CH_3_ and ZIF-8_Br.

The as-obtained ZIFs were analyzed using X-ray diffraction to verify the identity of the materials and assess their purity. Their respective XRD patterns were compared to those generated from their corresponding CIF files (Figure 2a). All experimental XRD patterns (peak position and relative intensity) matched those generated, confirming the success of the synthesis. The identification of all products was also confirmed using IR spectroscopy [54] (Tables S1 and S2). The IR spectra of ZIF-8_CH_3_ and ZIF-8_Br present strong similarities due to their isostructural characteristics (Figure 2b). However, the position of the IR bands differs slightly due to the effects of the chemical group grafted onto the imidazolate ligands. Thermogravimetric analysis of the two samples indicates that their thermal stability under airflow is similar even though ZIF-8_Br appears to be slightly more thermally robust. We can observe an initial slow mass loss from ~200 °C to ~300 °C followed by two more rapid decreases. This difference in kinetics can be explained by the nature of their degradation mechanism. The first step consists of the carbonization of the ligand [62] involving loss of the functional group (-CH_3_ or -Br), cleavage of Zn-N and C-N bonds, and subsequent formation of an imidazole-aziridine structure. Above 300 °C, the resulting materials undergo oxidation leading to the formation of residual ZnO at ~ 600 °C. The specific surface area (SA) and pore volume (V_p_) determined using N_2_ physisorption correspond to those generally reported in the literature (1633 vs. 1696 m^2^/g [61] and 670 vs. 660 m^2^/g [54]) for ZIF-8_CH_3_ and ZIF-8_Br, respectively. Interestingly, these experimental values also match those calculated theoretically (Table 1). As noted in the Section 3, both ZIFs were washed with alcohol after the synthesis and then deposited on the quartz support from alcohol suspension. The successful removal of alcohols from the MOF structure during the activation process thus confirms their regenerability and potential reuse for repetitive adsorption cycles.

### 2.3. Alcohol Adsorption and Separation

#### 2.3.1. Experimental Adsorption Measurement

**Figure 3:**
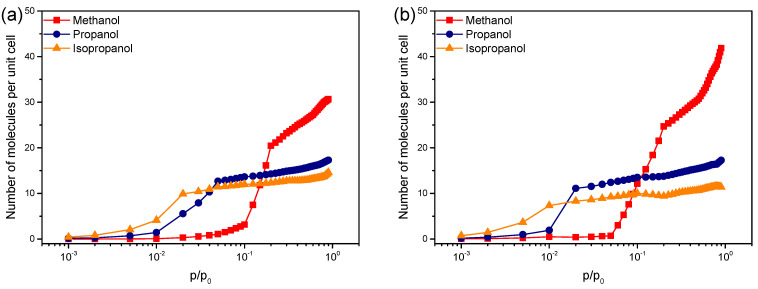
Alcohol adsorption isotherms obtained by QCM for (**a**) ZIF-8_CH_3_ and (**b**) ZIF-8_Br.

As detailed in the experimental part of the manuscript, the adsorption isotherms were obtained at 293K by QCM measurements with ZIF-8_CH_3_ and ZIF-8_Br exposed to either primary (methanol, n-propanol) or secondary (isopropanol) alcohol vapors (Figure 3). For both ZIFs, as the alkyl chain length of linear alcohols increases, the partial pressure threshold at which adsorption begins progressively shifts downward. This suggests that longer-chain alcohols interact more favorably with the ZIF, resulting in adsorption at lower partial pressure conditions. Comparison between C_3_ alcohol isomers (n-propanol and isopropanol) shows that isopropanol tends to be strongly adsorbed at very lower partial pressures (steeper isotherm slope for ZIF-8_Br at p/p_0_ < 0.01). The lower total adsorption capacity of ZIF-8_Br could be attributed concomitantly to isopropanol molecular rigidity and steric hindrance which limit its ability to fully occupy all available pore space of the MOF structure. Consequently, compared to n-propanol, isopropanol occupies the smaller ZIF-8_Br cavities (10.5 Å) less effectively than those in ZIF-8_CH_3_ (11.4 Å) resulting in faster saturation of the ZIF-8_Br porous structure. According to their initial affinity for ZIF-8 structure, the tested alcohols can be ranked as follows: isopropanol > n-propanol > methanol. The alcohols tested can then be ranked according to their affinity for the ZIF-8 structure: isopropanol > n-propanol > methanol. This order can be explained by the notable hydrophobicity of ZIF-8_CH_3_ which favors the preferential adsorption of low-polarity adsorbates. These results, therefore, clearly indicate that the adsorption process is mainly governed by the alcohol polarity. Such a conclusion is consistent with studies reported in the literature [51,53]. These findings were supported by periodic DFT calculations conducted to determine the positioning of the adsorbate in the ZIF-8_CH_3_ cavities. It appears that alcohols tend to localize preferentially near the 6-membered ring (6MR) windows, thereby maximizing their interactions with the framework structure (Figure S7). Specifically, methanol’s methyl and hydroxyl groups were observed to interact with ZIF ligands via their -CH_3_ and aromatic -C-H groups. In addition, the number of interactions increases proportionally to the lengthening of the aliphatic chain of the alcohol. This phenomenon favors the accommodation of alcohols within the ZIF cavity, thus corroborating the observed trend and the ranking of alcohols according to their molecular structure.

The adsorption behavior of ZIF-8_Br is distinguished by a notable shift of its isotherms towards lower partial pressures in comparison to ZIF-8_CH_3_ (Figure 3b). This result points out a higher affinity of the alcohols towards the former ZIF analog despite the similar sequence of alcohol adsorption. ZIF-8_Br is thus more performable for adsorbing all tested alcohols at lower partial pressures compared to ZIF-8_CH_3_. This finding is of great practical importance since typical concentrations of VOCs in an atmospheric environment are only around the range of a few ppb-ppm. It can, therefore, be concluded that the adsorption properties of ZIF-8_Br are more suitable for air purification or alcohol detection under realistic conditions.

#### 2.3.2. Computational Study of Adsorption Mechanisms

**Figure 4:**
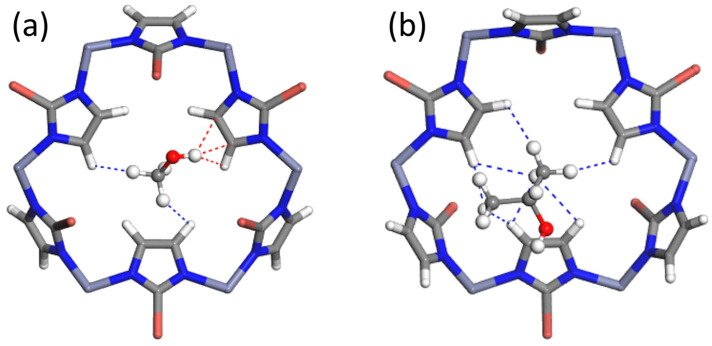
Positions of a single guest molecule of (**a**) methanol and (**b**) isopropanol, per unit cell in the ZIF-8_Br structure determined by DFT optimization. For better clarity, only the 6MR window closest to each alcohol molecule is shown. Only host–guest interactions at distances smaller than 3 Å were considered.

In order to elucidate the adsorption mechanism, the DFT approach was used to localize the adsorbate in the ZIF cavities and to identify host–guest interactions and their strength. In the case of ZIF-8_Br, DFT calculations show that alcohol molecules tend to localize near the 6MR windows (Figure 4) as already observed for ZIF-8_CH_3_ (Figure S7). However, a significant difference is observed in the increased contribution of the ZIF ligands in maintaining interactions with the adsorbates. DFT calculations reveal that these interactions mainly involve the C-H bonds of 2-bromoimidazolate ligands and hydroxyl groups as well as the aliphatic chains of alcohols.

The adsorption enthalpy at infinite dilution, also referred to as binding energy, was determined for each ZIF-alcohol pair through GCMC simulations, aiming to estimate the strength of adsorbent-adsorbate interactions (Table 2). The results highlight a correlation between the increase in the length of the hydrocarbon chain in alcohol and the enhanced interaction strength. The adsorption enthalpy values for ZIF-8_CH_3_ correspond to those reported in the literature [63], while a strong contrast is observed when comparing them with those of its halogenated derivative. In fact, the ZIF-8_Br displays almost double |∆H_ads_| values indicating stronger host–guest interactions and thus better adsorption properties of alcohols at lower partial pressures than in the case of ZIF-8_CH_3_. This suggests that additional interactions may contribute to the increased adsorption enthalpy. Halogen bonds, which can be stronger than other weak noncovalent interactions such as π–π stacking or dipole–dipole interactions, contribute to enhancing the overall adsorption enthalpy [64]. Furthermore, halogen groups are often associated with hydrophobic characteristics, which may explain the high adsorption enthalpy observed for the hydrophobic alcohols, n-propanol, and isopropanol. This result thus reaffirms the findings obtained by QCM measurements and DFT calculations, corroborating the potential of ZIF-8_Br for the adsorption of non-aromatic/aliphatic molecules.

The reported binding energy values for aliphatic alcohols in MOF structures are still very scarce, making comparisons with existing data difficult. Yet, Jiang et al. [63] theoretically calculated the binding energies of short linear alcohols (C_1_-C_4_) in ZIF-8_CH_3_ using the GCMC method, and these match well with the calculated adsorption enthalpies reported in our work. In the same study, the evolution of ∆H_ads_ as a function of vapor pressure was also computed, considering both host–guest interactions (at infinite dilution) and guest–guest interactions (number of molecules per unit cell > 1). Jiang’s study was validated by the experimental data of Kapteijn et al. [65], particularly for the adsorption of methanol using ZIF-8_CH_3_. Therefore, the good agreement between the theoretical results of our work and those of Jiang and Kapteijn confirms their accuracy and the reliability of the present computational outcomes.

Interestingly, according to Kapteijn et al. [65], ZIF-8_CH_3_ showed the best performance among 18 other MOFs for designing methanol-fueled adsorption-driven heat pumps. However, in our work, it appears that ZIF-8_Br exhibits superior performance to ZIF-8_CH_3_, demonstrating its potential for applications related to the adsorption and detection of short alcohols.

#### 2.3.3. IAST Selectivity for Alcohol Mixtures

**Figure 5:**
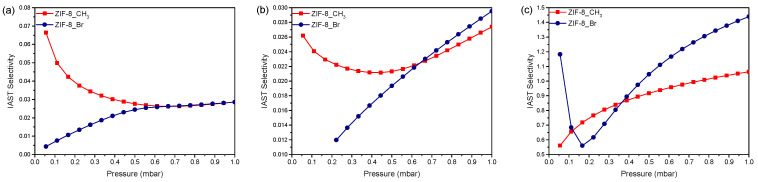
IAST selectivity as a function of partial pressures ranging from 0.0 to 1.0 mbar calculated for ZIF-8_CH_3_ (in red) and ZIF-8_Br (in blue) for three binary equimolar alcohol mixtures at 293K: (**a**) methanol/n-propanol, (**b**) methanol/isopropanol, and (**c**) n-propanol/isopropanol.

The adsorption performance of ZIF-8_CH_3_ and ZIF-8_Br was then investigated using the Ideal Adsorbed Solution Theory (IAST) to evaluate the material selectivity for binary equimolar alcohol mixtures at low pressures (0–1 mbar) and room temperature 293 K (Figure 5). This approach provides important insights into competitive adsorption behavior in mixed systems, which is essential for practical applications, where different types of alcohol may coexist.

For the methanol/n-propanol mixture, ZIF-8_CH_3_ exhibits a higher initial selectivity towards methanol (~0.065) when compared with ZIF-8_Br (~0.005) at 0.05 mbar. However, with increasing pressure, the selectivity of ZIF-8_CH_3_ for methanol gradually decreases, while that of ZIF-8_Br increases. At 1 mbar, both ZIFs converge to a selectivity of around 0.03. Importantly, ZIF-8_Br demonstrates superior discrimination between methanol and n-propanol at pressures below 0.6 mbar.

Similarly, in the case of methanol/isopropanol mixtures, ZIF-8_CH_3_ shows higher initial selectivity for methanol (0.026 at 0.05 mbar) in comparison with ZIF-8_Br (0.012 at 0.2 mbar). The selectivity of ZIF-8_CH_3_ decreases with increasing pressure, reaching a minimum of ~0.4 mbar. Conversely, the selectivity of ZIF-8_Br to methanol increases continuously, reaching ~0.030 at 1.0 mbar. This trend suggests that the difference in steric hindrance between methanol and isopropanol favors inevitably methanol adsorption at higher pressures despite the higher affinity of isopropanol for ZIF structures.

For n-propanol/isopropanol pairs, the separation efficiency is less pronounced, with selectivities fluctuating around 1 in the 0-1 mbar pressure range for both materials. ZIF-8_CH_3_ shows an initial selectivity of 0.55, increasing to 1.0 at 1.0 mbar. ZIF-8_Br displays more complex behavior, with an initial selectivity of 1.2, decreasing to 0.55 at 0.15 mbar, and then increasing again to 1.4 at 1.0 mbar. These observations indicate that, despite a lower affinity of n-propanol to ZIF-8_Br structure deduced from the pure-phase adsorption measurements, it is preferentially adsorbed when mixed with its bulkier branched isomer due to reduced steric hindrance effects.

To sum up, the C_3_ alcohol isomers (n-propanol and isopropanol) are preferentially adsorbed over methanol in equimolar mixtures, which is consistent with the findings obtained from the single-component adsorption isotherms. The IAST selectivity data show that ZIF-8_Br exhibits the most significant selectivity values for all studied alcohol pairs, particularly at low pressures. These results, complementary to those obtained from the pure-phase adsorption measurements, provide valuable insights into the competitive adsorption behavior of the studied MOFs and thus their potential for air purification and sensing applications. It could be, therefore, concluded that ZIF-8_Br presents promising properties for environmental remediation and the protection of human health.

## 3. Experimental Section

### 3.1. Materials and Methods

All reagents were purchased from commercial suppliers and used without any further purification. The synthesized materials were characterized using powder X-ray diffraction (PXRD), N_2_ sorption, Thermogravimetric analysis (TGA), and Fourier-transformed infrared (FTIR) spectroscopy. PXRD patterns were recorded using Malvern Panalytical X-Pert PRO (Cu Kα radiation, 45 kV, 25 mA) from 5 to 50° (2θ). N_2_ adsorption-desorption isotherms were obtained at 77 K (liquid nitrogen) using ASAP 2020 (Micromeritics). Samples were outgassed for 12 h at 200 °C under vacuum. Thermogravimetric analyses were carried out with TA instruments SDT 2960 under dry air at a constant heating rate of 5 °C/min from 25 to 800 °C. FTIR spectra were recorded from 600 to 4000 cm^−1^ (resolution of 4 cm^−1^) with Nicolet Nexus FT-IR apparatus in reflection mode.

### 3.2. Synthesis of ZIF-8_CH_3_ and ZIF-8_Br

ZIF-8_CH_3_ was synthesized according to the protocol reported elsewhere [61]. Typically, a solution of Zn(NO_3_)_2_.6H_2_O (9.9 mmol) in 200 mL of methanol was quickly poured into a solution of 2-methylimidazole (79.0 mmol) in 200 mL of methanol under stirring at room temperature. After 1 h of reaction, the solution was centrifuged at 9000 rpm for 30 min and the solid obtained was then redispersed in ethanol to eliminate excess reagents. This washing procedure was repeated three times.

ZIF-8_Br was prepared by mixing a solution of DMF (5 mL) containing Zn(CH_3_COO)_2_·2H_2_O (0.5 mmol) and a solution of methanol (5 mL) containing 2-bromoimidazole (1.0 mmol) at room temperature. The mixture was then left to stir for 1 h at room temperature. Afterward, the solution was centrifugated at 9000 rpm for 30 min, and the resulting solid was redispersed in ethanol. This step was repeated three times to eliminate unreacted species.

### 3.3. VOC Sorption Measurements Using QCM

Alcohol adsorption isotherms were obtained using a quartz crystal microbalance (OpenQCM) equipped with quartz crystals (5 MHz, purchased from Novaetech Srl, Napoli, Italy) coated with ZIF-8_CH_3_ or ZIF-8_Br. The ZIF overlayer was formed by evaporating 10 µL of an ethanolic suspension of ZIF particles (5 mg/mL) on the quartz support followed by its activation under vacuum at 150 °C for 2 h. The frequency variation before and after MOF deposition was inserted into the Sauerbrey equation [66] to deduce the overall mass deposited on the quartz surface. Immediately after the activation treatment at 150 °C for 2 h, the sample was cooled in the QCM chamber under a flow of dry N_2_. After 15 min stabilization at 20 °C, an alcohol vapor (methanol, n-propanol, or isopropanol) diluted in N_2_ was introduced into the QCM chamber. For each alcohol, the vapor partial pressure was varied from 0 to 0.9 P/P_0_ (where P_0_ is saturation pressure). The induced frequency variation of quartz was recorded in situ to obtain the mass uptake isotherm of the alcohol sorbed by the ZIF materials.

### 3.4. Computational Methods

Computational simulations were carried out to gain insights into the adsorption phenomena and to deduce the thermodynamic properties of the three specific alcohol-ZIF systems. ZIF-8_CH_3_ (CCDC deposit number: 739168) and ZIF-8_Br (CCDC deposit number: 1860456) structures were first cleaned of any presence of solvent molecules.

The structural characteristics of the ZIF structures, including pore limiting diameter (PLD), largest cavity diameter (LCD), theoretical specific surface area (SA), and pore volume (V_p_) were calculated using Zeo++ software [67].

The assignment of partial charges (Mulliken) on MOF structures was executed by periodic DFT using the CASTEP simulation package [68]. This approach was chosen since it captures the periodic nature of MOFs. The Hamiltonian operator was estimated by employing the Perdew–Burke–Ernzerhof (PBE) exchange-correlation functional and the Tkatchenko–Scheffler dispersion correction. The molecular wavefunction description was obtained using “on-the-fly” pseudopotentials and a plane wave basis set operating at 570 eV, which gave convergence to within 5 × 10^−7^ eV per atom. The potential energy surface was searched for energy minima by means of the Broyden–Fletcher–Goldfarb–Shanno (BFGS) algorithm.

The ZIF structures bearing the Mulliken charges previously determined by periodic DFT were prepared as 2 × 2 × 2 supercells with lengths larger than at least twice the Van der Walls cutoff value (14 Å). Atoms were represented using a simplified model, where each of them was treated as a single Lennard-Jones (L-J) interaction site. Their interactions were described using the Universal Force Field (UFF) and Dreiding Force Field. Host–guest interactions were modeled using the L-J potential. The Lorentz–Berthelot mixing rules were applied to determine the parameters for these L-J interactions. To accurately account for the long-range electrostatic interactions, the Ewald summation method was employed. This approach ensured a relative accuracy of 10^−6^ for the calculation of the electrostatic forces and energies. By combining the Lennard-Jones potential with the Ewald summation technique, the model was able to comprehensively capture the short-range van der Waals interactions as well as the long-range electrostatic effects between host and guest species.

Each alcohol molecule was represented using uncharged united-atom TraPPE (TraPPE-UA) models [69]. Typically, in these models, CH_x_ groups (e.g., methyl, methylene, or aromatic CH) are treated as pseudo-atoms located at the carbon atom sites, while all other atoms (e.g., O and H from hydroxyl groups) are modeled explicitly. The models for alcohol molecules are described in more detail in SI (Figure S4).

The estimation of adsorption enthalpy values at infinite dilution (also denoted as binding energy), ∆H_ads_, was obtained by executing Gran Canonical Monte Carlo (GCMC) simulations and was deduced from the following equation [70]:

∆H = <U_hg_> − <U_h_> − <U_g_> − RT

where <U_hg_> represents the average energy of the guest molecule within the host framework, <U_h_> the average energy of the host framework, and <U_g_> denotes the average energy of a single guest molecule in the gas phase. The term RT represents the enthalpy per particle of the ideal bulk phase.

For these simulations, the number of cycles was set to 10,000,000 to reach equilibrium (Figure S5) and run at 293 K under atmospheric pressure.

The simulated annealing method was employed to determine the lowest energy configuration of alcohol molecules (one per unit cell) in the primitive cells of the two considered ZIF structures. Additionally, the configurational-bias Monte Carlo technique was used to identify the minimum energy configuration in each scenario. During these simulations, the framework atoms remained fixed. Both mutual interactions between gas molecules themselves and the adsorbent were modeled using a combination of Lennard-Jones (LJ) and Coulombic potentials. The LJ potential parameters for the framework atoms and adsorbates were obtained from the Universal Force Field, while the charges for the framework atoms were determined using the charge equilibration (Q_eq_) method.

The preferential location of an adsorbate molecule within each ZIF structure was then refined by geometry optimization using the CASTEP package from the lowest energy ZIF-alcohol configuration previously obtained using the simulated annealing method. The parameters remained similar to the charge assignment process. The ZIF-adsorbate systems were considered as optimized when the energy per atom, maximum force, maximum stress, and maximum atomic displacement converged to the values of 2 × 10^−5^ kcal/mol, 0.001 eV/Å, 0.001 GPa, and 1 × 10^−5^ Å, respectively.

The Ideal Adsorbed Solution Theory (IAST) was used to investigate the adsorption performance of materials from the single-component adsorption isotherms. The latter were fitted with Dual-Site Langmuir-Freundlich using IAST++ software (version 1.0.1.). The selectivity (S) was obtained through the following formula:

$$S_{A/B}=\frac{q_{A}}{y_{A}}\times \frac{y_{B}}{q_{B}}\;\;$$

where *S_A/B_* represents the selectivity of adsorption for component A relative to component B, *q_A_* and *q_B_* denote the amounts adsorbed at equilibrium for components A and B and *y_A_* and *y_B_* correspond to the molar fractions of components A and B in the gas-phase mixture.

## 4. Conclusions

In summary, the present study focused on the attractive adsorption properties of ZIF-8_Br for capturing harmful aliphatic alcohols (methanol, n-propanol, and isopropanol). Compared to the iconic ZIF-8_CH_3_, its halogenated analog ZIF-8_Br exhibits a higher affinity for aliphatic alcohols at lower partial pressures, making it a more suitable candidate for air purification or sensing applications under realistic conditions. Computational and experimental investigations have provided reliable insights into the adsorption mechanisms, revealing that the length of the hydrocarbon chain in alcohols and the presence of halogenated ligands in the ZIF structure significantly influence the adsorption capacity and the strength of interaction. Moreover, the Ideal Adsorbed Solution Theory was used to investigate the adsorption properties and selectivity in binary equimolar alcohol mixtures. The performance of ZIF-8_Br has been confirmed in the presence of interference molecules, thus reinforcing its potential also for selective sorption applications. This research lays the foundation for the development of effective adsorbents and sensors to monitor alcohol emissions at low partial pressures, thereby contributing to the protection of the environment and the population.

## Figures and Tables

**Table 1:** Calculated structural features of ZIF-8_CH_3_ and ZIF-8_Br including unit cell volume, pore limiting diameter (PLD), largest cavity diameter (LCD), surface area (SA), and pore volume (V_p_).

Structure	Unit Cell Volume (Å^3^)	PLD (Å)	LCD (Å)	SA (m^2^/g)	V_p_ (cm^3^/g)
ZIF-8_CH_3_	4904.42	3.42	11.4	1703	1.10
ZIF-8_Br	4985.25	3.39	10.5	660	0.31

**Table 2:** Values of ∆H_ads_ at infinite dilution (kJ/mol) determined using GCMC simulation for the studied series of aliphatic alcohols accommodated in ZIF-8_CH_3_ and ZIF-8_Br structures.

Adsorbent	∆H_ads_ (kJ/mol)		
	Methanol	n-Propanol	Isopropanol
ZIF-8_CH_3_	−22.9	−38.7	−41.2
ZIF-8_Br	−47.2	−63.5	−65.6

## Data Availability

The data presented in this study are available upon request from the corresponding author. The data are not publicly available due to the institution’s policy.

## Supplementary Materials

The following supporting information can be downloaded at: https://www.mdpi.com/article/10.3390/molecules29245825/s1, Figure S1: N2 adsorption/desorption isotherms of ZIF-8_CH3 and ZIF-8_Br. Figure S2: Thermogravimetric analysis of ZIF-8_CH_3_ and ZIF-8_Br. Figure S3: Partial charge assignments on the structure of (**a**) ZIF-8_CH_3_ and (**b**) ZIF-8_Br. Figure S4: Model representations of alcohol molecules: (**a**) methanol, (**b**) propanol, (**c**) isopropanol. Figure S5: Evolution of adsorption energy accuracy according to the number of GCMC production cycles. Figure S6: Interactions between propanol and ZIF-8_Br structure. Figure S7: Host–guest interactions determined by periodic DFT for systems composed of ZIF-8 structure and (**a**) methanol, (**b**) n-propanol, and (**c**) isopropanol adsorbates. Table S1: IR absorption band assignments of ZIF-8_CH_3_. Table S2: IR absorption band assignments of ZIF-8_Br. Table S3: Force Field parameters for MOF structures.

## References

[1] Gakidou E., Afshin A., Abajobir A.A., Abate K.H., Abbafati C., Abbas K.M., Abd-Allah F., Abdulle A.M., Abera S.F., Aboyans V. (2017). Global, Regional, and National Comparative Risk Assessment of 84 Behavioural, Environmental and Occupational, and Metabolic Risks or Clusters of Risks, 1990–2016: A Systematic Analysis for the Global Burden of Disease Study 2016. Lancet.

[2] Rumchev K. (2004). Association of Domestic Exposure to Volatile Organic Compounds with Asthma in Young Children. Thorax.

[3] Pappas G.P., Herbert R.J., Henderson W., Koenig J., Stover B., Barnhart S. (2000). The Respiratory Effects of Volatile Organic Compounds. Int. J. Occup. Environ. Health.

[4] Tang L., Liu M., Tian J. (2024). Volatile Organic Compounds Exposure Associated with Depression among U.S. Adults: Results from NHANES 2011–2020. Chemosphere.

[5] Zheng J., Pang Y., Zhang Y., Hu W., Yang P., Liu Q., Ning J., Du Z., Jin X., Tang J. (2022). Indoor VOCs Exposure Induced Parkinson-like Behaviors through Autophagy Dysfunction and NLRP3 Inflammasome-Mediated Neuroinflammation. J. Hazard. Mater..

[6] Hussain M.S., Gupta G., Mishra R., Patel N., Gupta S., Alzarea S.I., Kazmi I., Kumbhar P., Disouza J., Dureja H. (2024). Unlocking the Secrets: Volatile Organic Compounds (VOCs) and Their Devastating Effects on Lung Cancer. Pathol.-Res. Pract..

[7] Xiong Y., Du K., Huang Y. (2024). One-Third of Global Population at Cancer Risk Due to Elevated Volatile Organic Compounds Levels. npj Clim. Atmos. Sci..

[8] Chen Y., Shi Y., Ren J., You G., Zheng X., Liang Y., Simayi M., Hao Y., Xie S. (2023). VOC Species Controlling O3 Formation in Ambient Air and Their Sources in Kaifeng, China. Environ. Sci. Pollut. Res..

[9] Kim S.-Y., Kim E., Kim W.J. (2020). Health Effects of Ozone on Respiratory Diseases. Tuberc. Respir. Dis..

[10] Patočka J., Kuča K. (2012). Toxic Alcohols: Aliphatic Saturated Alcohols. Mil. Med. Sci. Lett..

[11] Nekoukar Z., Zakariaei Z., Taghizadeh F., Musavi F., Banimostafavi E.S., Sharifpour A., Ghuchi N.E., Fakhar M., Tabaripour R., Safanavaei S. (2021). Methanol Poisoning as a New World Challenge: A Review. Ann. Med. Surg..

[12] Tasar R., Wiegand C., Elsner P. (2021). How Irritant Are N-propanol and Isopropanol?—A Systematic Review. Contact Dermat..

[13] Burleigh-Flayer H. (1997). Isopropanol Vapor Inhalation Oncogenicity Study in Fischer 344 Rats and CD-1 Mice. Fundam. Appl. Toxicol..

[14] Yamamoto T., Kim Y.H., Kim B.C., Endo A., Thongprachan N., Ohmori T. (2012). Adsorption Characteristics of Zeolites for Dehydration of Ethanol: Evaluation of Diffusivity of Water in Porous Structure. Chem. Eng. J..

[15] Gunst D., Alexopoulos K., Van Der Borght K., John M., Galvita V., Reyniers M.-F., Verberckmoes A. (2017). Study of Butanol Conversion to Butenes over H-ZSM-5: Effect of Chemical Structure on Activity, Selectivity and Reaction Pathways. Appl. Catal. A Gen..

[16] Lu W., Wei Z., Gu Z.Y., Liu T.F., Park J., Park J., Tian J., Zhang M., Zhang Q., Gentle T. (2014). Tuning the Structure and Function of Metal-Organic Frameworks via Linker Design. Chem. Soc. Rev..

[17] Zhang X., Chen Z., Liu X., Hanna S.L., Wang X., Taheri-Ledari R., Maleki A., Li P., Farha O.K. (2020). A Historical Overview of the Activation and Porosity of Metal–Organic Frameworks. Chem. Soc. Rev..

[18] Forrest K.A., Pham T., Elsaidi S.K., Mohamed M.H., Thallapally P.K., Zaworotko M.J., Space B. (2019). Investigating CO_2_ Sorption in SIFSIX-3-M (M = Fe, Co, Ni, Cu, Zn) through Computational Studies. Cryst. Growth Des..

[19] Dedecker K., Drobek M., Rouessac V., Julbe A. (2023). A Palladium-Based MOF for the Preferential Sorption of Benzene. ACS Appl. Mater. Interfaces.

[20] Van De Voorde B., Hezinová M., Lannoeye J., Vandekerkhove A., Marszalek B., Gil B., Beurroies I., Nachtigall P., De Vos D. (2015). Adsorptive Desulfurization with CPO-27/MOF-74: An Experimental and Computational Investigation. Phys. Chem. Chem. Phys..

[21] Pires J., Fernandes J., Dedecker K., Gomes J.R.B., Pérez-Sánchez G., Nouar F., Serre C., Pinto M.L. (2019). Enhancement of Ethane Selectivity in Ethane-Ethylene Mixtures by Perfluoro Groups in Zr-Based Metal-Organic Frameworks. ACS Appl. Mater. Interfaces.

[22] Dedecker K., Pillai R.S., Nouar F., Pires J., Steunou N., Dumas E., Maurin G., Serre C., Pinto M.L. (2018). Metal-Organic Frameworks for Cultural Heritage Preservation: The Case of Acetic Acid Removal. ACS Appl. Mater. Interfaces.

[23] Yaghi O.M. (2008). High-Throughput Synthesis of Zeolitic. ReVision.

[24] Padial N.M., Quartapelle Procopio E., Montoro C., López E., Oltra J.E., Colombo V., Maspero A., Masciocchi N., Galli S., Senkovska I. (2013). Highly Hydrophobic Isoreticular Porous Metal–Organic Frameworks for the Capture of Harmful Volatile Organic Compounds. Angew. Chem. Int. Ed..

[25] Dedecker K., Drobek M., Julbe A. (2023). Effect of Ligand Aromaticity on Cyclohexane and Benzene Sorption in IRMOFs: A Computational Study. J. Phys. Chem. B.

[26] Dedecker K., Dumas E., Lavédrine B., Steunou N., Serre C. (2019). 5—Metal-Organic Frameworks for the Capture of Volatile Organic Compounds and Toxic Chemicals. Metal-Organic Frameworks (MOFs) for Environmental Applications.

[27] Dasgupta S., Biswas S., Dedecker K., Dumas E., Menguy N., Berini B., Lavedrine B., Serre C., Boissière C., Steunou N. (2023). In Operando Spectroscopic Ellipsometry Investigation of MOF Thin Films for the Selective Capture of Acetic Acid. ACS Appl. Mater. Interfaces.

[28] Gao C., Zou P., Ji S., Xing Y., Cai J., Wu J., Wu T. (2023). High-Flux Loose Nanofiltration Membrane with Anti-Dye Fouling Ability Based on TA@ZIF-8 for Efficient Dye/Salt Separation. J. Environ. Chem. Eng..

[29] Li X.-Y., Li Y.-Z., Ma L.-N., Hou L., He C.-Z., Wang Y.-Y., Zhu Z. (2020). Efficient Gas and Alcohol Uptake and Separation Driven by Two Types of Channels in a Porous MOF: An Experimental and Theoretical Investigation. J. Mater. Chem. A.

[30] Xu L.-H., Li S.-H., Mao H., Li Y., Zhang A.-S., Wang S., Liu W.-M., Lv J., Wang T., Cai W.-W. (2022). Highly Flexible and Superhydrophobic MOF Nanosheet Membrane for Ultrafast Alcohol-Water Separation. Science.

[31] He T., Kong X.-J., Bian Z.-X., Zhang Y.-Z., Si G.-R., Xie L.-H., Wu X.-Q., Huang H., Chang Z., Bu X.-H. (2022). Trace Removal of Benzene Vapour Using Double-Walled Metal–Dipyrazolate Frameworks. Nat. Mater..

[32] Han Y., Huang W., He M., An B., Chen Y., Han X., An L., Kippax-Jones M., Li J., Yang Y. (2024). Trace Benzene Capture by Decoration of Structural Defects in Metal–Organic Framework Materials. Nat. Mater..

[33] Liu M., Cai Y., Liu Q., Jin X., Xue C., Zhang S., Feng P., Luo Y. (2024). Porous Calcium-Silicate-Hydrate as a Low-Cost Nano-Platform for Ultra-High CO_2_ Capture and Storage. Small Methods.

[34] Li S., Sun Y., Wang Z., Jin C., Yin M., An Q. (2023). Rapid Fabrication of High-Permeability Mixed Matrix Membranes at Mild Condition for CO_2_ Capture. Small.

[35] Phan A., Doonan C.J., Uribe-Romo F.J., Knobler C.B., Okeeffe M., Yaghi O.M. (2010). Synthesis, Structure, and Carbon Dioxide Capture Properties of Zeolitic Imidazolate Frameworks. Acc. Chem. Res..

[36] Yaghi O.M., O’Keeffe M., Ockwig N.W., Chae H.K., Eddaoudi M., Kim J. (2003). Reticular Synthesis and the Design of New Materials. Nature.

[37] Park K.S., Ni Z., Côté A.P., Choi J.Y., Huang R., Uribe-Romo F.J., Chae H.K., O’Keeffe M., Yaghi O.M. (2006). Exceptional Chemical and Thermal Stability of Zeolitic Imidazolate Frameworks. Proc. Natl. Acad. Sci. USA.

[38] Coudert F. (2017). Molecular Mechanism of Swing Effect in Zeolitic Imidazolate Framework ZIF-8: Continuous Deformation upon Adsorption. ChemPhysChem.

[39] Fairen-Jimenez D., Moggach S.A., Wharmby M.T., Wright P.A., Parsons S., Düren T. (2011). Opening the Gate: Framework Flexibility in ZIF-8 Explored by Experiments and Simulations. J. Am. Chem. Soc..

[40] Aguado S., Bergeret G., Pera-Titus M., Moizan V., Nieto-Draghi C., Bats N., Farrusseng D. (2011). Guest-Induced Gate-Opening of a Zeolite Imidazolate Framework. New J. Chem..

[41] Arami-Niya A., Birkett G., Zhu Z., Rufford T.E. (2017). Gate Opening Effect of Zeolitic Imidazolate Framework ZIF-7 for Adsorption of CH_4_ and CO_2_ from N_2_. J. Mater. Chem. A.

[42] Krokidas P., Moncho S., Brothers E.N., Castier M., Economou I.G. (2018). Tailoring the Gas Separation Efficiency of Metal Organic Framework ZIF-8 through Metal Substitution: A Computational Study. Phys. Chem. Chem. Phys..

[43] Loloei M., Kaliaguine S., Rodrigue D. (2022). CO_2_-Selective Mixed Matrix Membranes of Bimetallic Zn/Co-ZIF vs. ZIF-8 and ZIF-67. Sep. Purif. Technol..

[44] Mor J., Nelliyil R.B., Sharma S.K. (2023). Fine-Tuning of the Pore Aperture and Framework Flexibility of Mixed-Metal (Zn/Co) Zeolitic Imidazolate Framework-8: An In Situ Positron Annihilation Lifetime Spectroscopy Study under CO_2_ Gas Pressure. Langmuir.

[45] Han C., Zhang C., Tymińska N., Schmidt J.R., Sholl D.S. (2018). Insights into the Stability of Zeolitic Imidazolate Frameworks in Humid Acidic Environments from First-Principles Calculations. J. Phys. Chem. C.

[46] Zheng B., Wang L.L., Du L., Huang K.-W., Du H. (2016). ZIF-8 Gate Tuning via Terminal Group Modification: A Computational Study. Chem. Phys. Lett..

[47] Pokhrel J., Bhoria N., Anastasiou S., Tsoufis T., Gournis D., Romanos G., Karanikolos G.N. (2018). CO_2_ Adsorption Behavior of Amine-Functionalized ZIF-8, Graphene Oxide, and ZIF-8/Graphene Oxide Composites under Dry and Wet Conditions. Microporous Mesoporous Mater..

[48] Salles F., Zajac J. (2021). Impact of Structural Functionalization, Pore Size, and Presence of Extra-Framework Ions on the Capture of Gaseous I2 by Mof Materials. Nanomaterials.

[49] Ortiz A.U., Freitas A.P., Boutin A., Fuchs A.H., Coudert F.-X. (2014). What Makes Zeolitic Imidazolate Frameworks Hydrophobic or Hydrophilic? The Impact of Geometry and Functionalization on Water Adsorption. Phys. Chem. Chem. Phys..

[50] Zhang K., Nalaparaju A., Chen Y., Jiang J. (2014). Biofuel Purification in Zeolitic Imidazolate Frameworks: The Significant Role of Functional Groups. Phys. Chem. Chem. Phys..

[51] Zhang K., Lively R.P., Dose M.E., Brown A.J., Zhang C., Chung J., Nair S., Koros W.J., Chance R.R. (2013). Alcohol and Water Adsorption in Zeolitic Imidazolate Frameworks. Chem. Commun..

[52] Khan A.H., Salout S.A., Shupletsov L., De A., Senkovska I., Kaskel S., Brunner E. (2022). Solid-State NMR Insights into Alcohol Adsorption by Metal–Organic Frameworks: Adsorption State, Selectivity, and Adsorption-Induced Phase Transitions. Chem. Commun..

[53] Yim C., Lee M., Kim W., Lee S., Kim G.-H., Kim K.T., Jeon S. (2015). Adsorption and Desorption Characteristics of Alcohol Vapors on a Nanoporous ZIF-8 Film Investigated Using Silicon Microcantilevers. Chem. Commun..

[54] Yagi R., Ueda T. (2023). Substitution (CH_3_, Cl, or Br) Effects of the Imidazolate Linker on Benzene Adsorption Kinetics for the Zeolitic Imidazolate Framework (ZIF)-8. Phys. Chem. Chem. Phys..

[55] Drobek M., Kim J.H., Bechelany M., Vallicari C., Julbe A., Kim S.S. (2016). MOF-Based Membrane Encapsulated ZnO Nanowires for Enhanced Gas Sensor Selectivity. ACS Appl. Mater. Interfaces.

[56] Weber M., Kim J.H., Lee J.H., Kim J.Y., Iatsunskyi I., Coy E., Drobek M., Julbe A., Bechelany M., Kim S.S. (2018). High-Performance Nanowire Hydrogen Sensors by Exploiting the Synergistic Effect of Pd Nanoparticles and Metal-Organic Framework Membranes. ACS Appl. Mater. Interfaces.

[57] Drobek M., Bechelany M., Vallicari C., Abou Chaaya A., Charmette C., Salvador-Levehang C., Miele P., Julbe A. (2015). An Innovative Approach for the Preparation of Confined ZIF-8 Membranes by Conversion of ZnO ALD Layers. J. Memb. Sci..

[58] Koo W.-T., Jang J.-S., Kim I.-D. (2019). Metal-Organic Frameworks for Chemiresistive Sensors. Chem.

[59] Tu M., Wiktor C., Rösler C., Fischer R.A. (2014). Rapid Room Temperature Syntheses of Zeolitic-Imidazolate Framework (ZIF) Nanocrystals. Chem. Commun..

[60] Öztürk Z., Filez M., Weckhuysen B.M. (2017). Decoding Nucleation and Growth of Zeolitic Imidazolate Framework Thin Films with Atomic Force Microscopy and Vibrational Spectroscopy. Chem.—A Eur. J..

[61] Demessence A., Boissière C., Grosso D., Horcajada P., Serre C., Férey G., Soler-Illia G.J.A.A., Sanchez C. (2010). Adsorption Properties in High Optical Quality NanoZIF-8 Thin Films with Tunable Thickness. J. Mater. Chem..

[62] James J.B., Lin Y.S. (2016). Kinetics of ZIF-8 Thermal Decomposition in Inert, Oxidizing, and Reducing Environments. J. Phys. Chem. C.

[63] Zhang K., Zhang L., Jiang J. (2013). Adsorption of C 1 –C 4 Alcohols in Zeolitic Imidazolate Framework-8: Effects of Force Fields, Atomic Charges, and Framework Flexibility. J. Phys. Chem. C.

[64] Cavallo G., Metrangolo P., Milani R., Pilati T., Priimagi A., Resnati G., Terraneo G. (2016). The Halogen Bond. Chem. Rev..

[65] de Lange M.F., van Velzen B.L., Ottevanger C.P., Verouden K.J.F.M., Lin L.-C., Vlugt T.J.H., Gascon J., Kapteijn F. (2015). Metal–Organic Frameworks in Adsorption-Driven Heat Pumps: The Potential of Alcohols as Working Fluids. Langmuir.

[66] Sauerbrey G. (1959). The Use of Quartz Oscillators for Weighing Thin Layers and for Microweighing. Z. Phys..

[67] Willems T.F., Rycroft C.H., Kazi M., Meza J.C., Haranczyk M. (2012). Algorithms and Tools for High-Throughput Geometry-Based Analysis of Crystalline Porous Materials. Microporous Mesoporous Mater..

[68] Kratzer P., Neugebauer J. (2019). The Basics of Electronic Structure Theory for Periodic Systems. Front. Chem..

[69] Chen B., Potoff J.J., Siepmann J.I. (2001). Monte Carlo Calculations for Alcohols and Their Mixtures with Alkanes. Transferable Potentials for Phase Equilibria. 5. United-Atom Description of Primary, Secondary, and Tertiary Alcohols. J. Phys. Chem. B.

[70] Woods G.B., Panagiotopoulos A.Z., Rowlinson J.S. (1988). Adsorption of Fluids in Model Zeolite Cavities. Mol. Phys..

