# Supplementary material for: Harnessing Halogenated Zeolitic Imidazolate Frameworks for Alcohol Vapor Adsorption
Source: Molecules. 2024 Dec 10;29(24):5825. doi: 10.3390/molecules29245825 (PMC11678290; doi:10.3390/molecules29245825)
Supplement: Supplementary file 1 [file molecules-29-05825-s001.zip › molecules-3349804-supplementary.pdf]

Supporting Information

Harnessing Halogenated Zeolitic Imidazolate Frameworks for Alcohol Vapor Adsorption

Kevin Dedecker*, Martin Drobek*, Anne Julbe

Institut Européen des Membranes (IEM); CNRS, ENSCM, Univ Montpellier; Place Eugène Bataillon; 34095 Montpellier; France

*Email: kevin.dedecker@umontpellier.fr and martin.drobek@umontpellier.fr

Materials characterization

N_2 adsorption-desorption isotherms were obtained at 77K (liquid nitrogen bath) using ASAP 2020 (Micromeritics). Samples were outgassed for 12h at 200°C under vacuum.

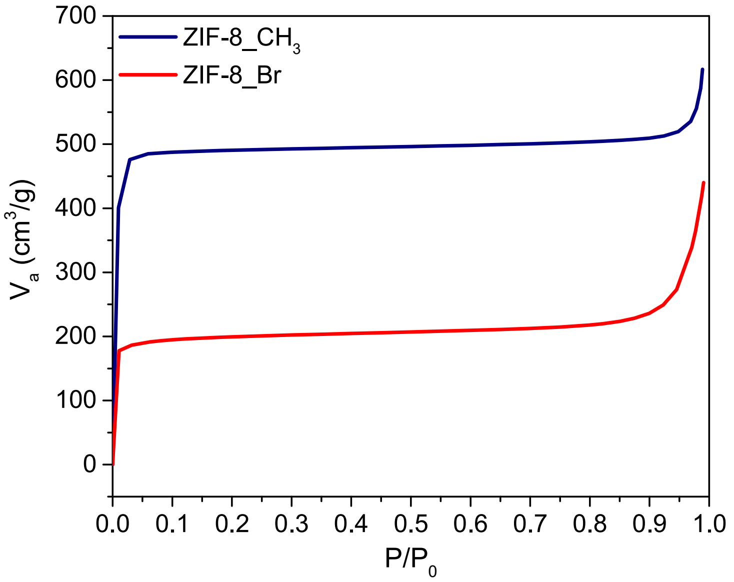

Figure S1: N_2 adsorption/desorption isotherms of ZIF-8_CH_3 and ZIF-8_Br.

Thermogravimetric analyses were carried out with TA instruments SDT 2960 under dry air at a constant heating rate of 5°C/min from 25 to 800°C.

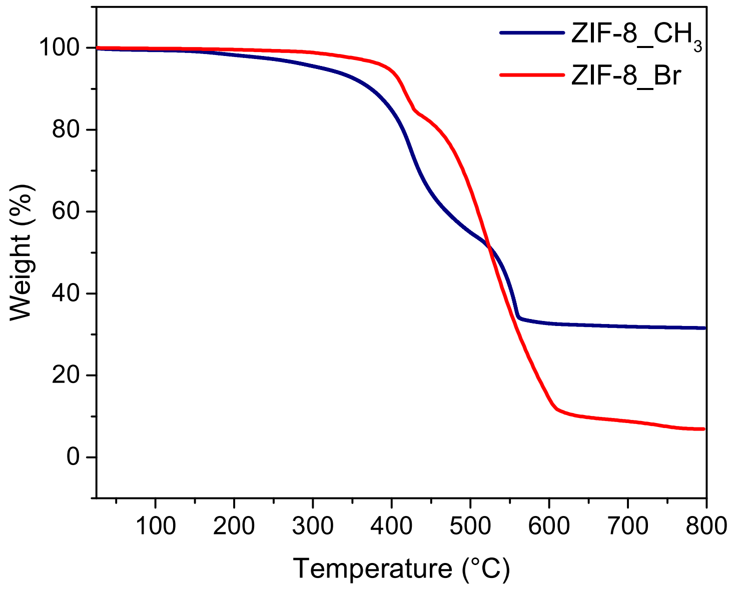

Figure S2: Thermogravimetric analysis of ZIF-8_CH_3 and ZIF-8_Br.

FTIR spectra were recorded from 600 to 4000 cm^{-1} (resolution of 4 cm^{-1}) with Nicolet Nexus FT-IR apparatus in reflection mode. The experimental values are compared with those reported in literature[1].

Table S1: IR absorption band assignments of ZIF-8_CH_3

IR band	Wavenumber (cm^{-1})	
	Reported values	This work
Methyl stretch + Ring puckering (S)	693	684
H_{ring} symmetric out-of-plane bend (S)	758	759
Ring in-plane bend (W/VW)	953	954
H_{methyl} bend (S)	995	995
H_{ring} wag (S)	1146	1146
Ring breathing (C-N symmetric stretch) (S)	1180	1180
H_{methyl} scissor(M)	1310	1311
Ring deformation (C-N asymmetric stretch) (S)	1382	1383
H_{methyl} bend (S)	1426	1424
C=C stretch + C_{ring}-C_{methyl} stretch (W)	1510	1510
Ring breathing (C=C stretch) (M)	1584	1584
H_{methyl} symmetric stretch (W)	2929	2931
H_{ring} symmetric stretch (M)	3134	3135

Table S2: IR absorption bands assignments of ZIF-8_Br

IR band	Wavenumber (cm^{-1})	
	Reported values	This work
Ring puckering (S)	675	676
H_{ring} symmetric out-of-plane bend (S)	749	751
H_{ring} symmetric out-of-plane bend (S)	834	835
H_{ring} symmetric out-of-plane bend (S)	952	955
H_{ring} symmetric out-of-plane bend (S)	976	977
H_{ring} wag (S)	1133	1135
H_{ring} wag (M)	1161	1163
Ring deformation (C-N asymmetric stretch) (S)	1352	1316
H_{ring} symmetric in-of-plane bend (W)	1440	1442
C=C stretch (W)	1576	1578
H_{ring} symmetric stretch (M)	3125	3125
H_{ring} symmetric stretch (M)	3144	3144

[1] R. Yagi and T. Ueda, Phys. Chem. Chem. Phys., 2023, 25, 20585–20596.

Computational details

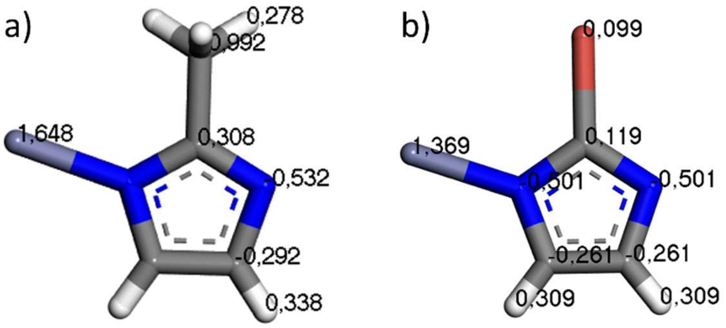

Figure S3: Partial charge assignments on the structure of a) ZIF-8_CH_3 and b) ZIF-8_Br.

Table S3: Force Field parameters for MOF structures

Type	Force Field	σ (A)	ε/kB (K)
**Zn**	UFF	2.46155	62.3992
**C**	Dreiding	3.47299	47.8562
**H**	Dreiding	2.84642	7.64893
**N**	Dreiding	3.03315	48.1581
**Br**	Dreiding	186.191	3.51905

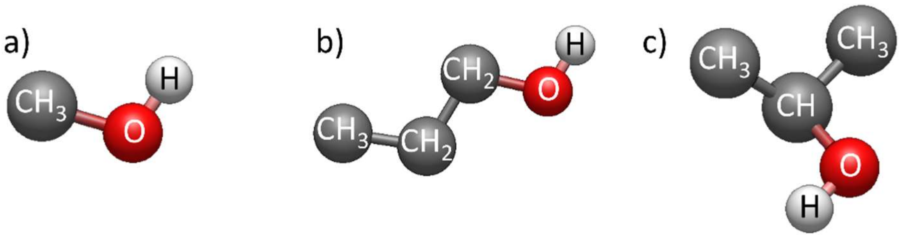

Figure S4: Model representations of alcohols molecules: a) methanol, b) propanol, c) isopropanol.

Methanol

Nonbonded interactions

#	(pseudo)atom	Type	ε/k_B [K]	σ [Å]	q [e]
1	CH_3	$[CH_3]$-O-H	98	3.75	0.265
2	O	CH_x-[O]-H	93	3.02	-0.700
3	H	O-[H]	0.0	0.0	0.435

1-2 bonded interactions

#	Stretch	Type	Length [Å]
1	1 – 2	CH_x-OH	1.43
2	2 – 3	O-H	0.945

1-3 bonded interactions

#	Bend	Type	θ [°]	k_θ/k_B [K/rad^2]
1	1 – 2 – 3	CHx-(O)-H	108.50	55400

n-Propanol

Nonbonded interactions

#	(pseudo)atom	Type	ε/k_B [K]	σ [Å]	q [e]
1	CH_3	$[CH_3]$-CHx	98	3.75	0.0
2	CH_2	CH_x-$[CH_2]$-CH_x	46	3.95	0.0
3	CH_2	CH_x-$[CH_2]$-O-H	46	3.95	0.265
4	O	CH_x-[O]-H	93	3.02	-0.700
5	H	O-[H]	0.0	0.0	0.435

1-2 Bonded Interactions

#	Stretch	Type	Length [Å]
1	1 – 2	CH_x-CH_y	1.54
2	2 – 3	CH_x-CH_y	1.54
3	3 – 4	CH_x-OH	1.43
4	4 – 5	O-H	0.945

1-3 Bonded Interactions

#	Bend	Type	θ [°]	k_θ/k_B [K/rad^2]
1	1 – 2 – 3	CH_x-(CH_2)-CH_y	114	62500
2	2 – 3 – 4	CH_x-(CH_y)-OH	109.47	50400
3	3 – 4 – 5	CH_x-(O)-H	108.50	55400

1-4 Bonded Interactions

#	Torsion	Type	c_0/k_B [K]	c_1/k_B [K]	c_2/k_B [K]	c_3/k_B [K]
1	1 – 2 – 3 – 4	CH_x-(CH_2)-(CH_2)-O	0.00	176.62	-53.34	769.93
2	2 – 3 – 4 – 5	CH_x-(CH_2)-(O)-H	0.00	209.82	-29.17	187.93

Isopropanol

Nonbonded interactions

#	(pseudo)atom	Type	ε/k_B [K]	σ [Å]	q [e]
1	CH_3	[CH_3]-CH_x	98	3.75	0.0
2	CH	(CH_x)$_2$-[CH]-O-H	10	4.33	0.265
3	CH_3	[CH_3]-CH_x	98	3.75	0.0
4	O	CH_x-[O]-H	93	3.02	-0.700
5	H	O-[H]	0.0	0.0	0.435

1-2 Bonded Interactions

#	Stretch	Type	Length [Å]
1	1 – 2	CH_x-CH_y	1.54
2	2 – 3	CH_x-CH_y	1.54
3	2 – 4	CH_x-OH	1.43
4	4 – 5	O-H	0.945

1-3 Bonded Interactions

#	Bend	Type	θ [°]	k_θ/k_B [K/rad^2]
1	1 – 2 – 3	CH_x-(CH)-CH_y	112	62500
2	1 – 2 – 4	CH_x-(CH_y)-OH	109.47	50400
3	2 – 4 – 5	CH_x-(O)-H	108.50	55400
4	3 – 2 – 4	CH_x-(CH_y)-OH	109.47	50400

1-4 Bonded Interactions

#	Torsion	Type	c_0/k_B [K]	c_1/k_B [K]	c_2/k_B [K]	c_3/k_B [K]
1	1 – 2 – 4 – 5	CHx-(CH)-(O)-H	215.89	197.33	31.46	-173.92
2	3 – 2 – 4 – 5	CHx-(CH)-(O)-H	215.89	197.33	31.46	-173.92

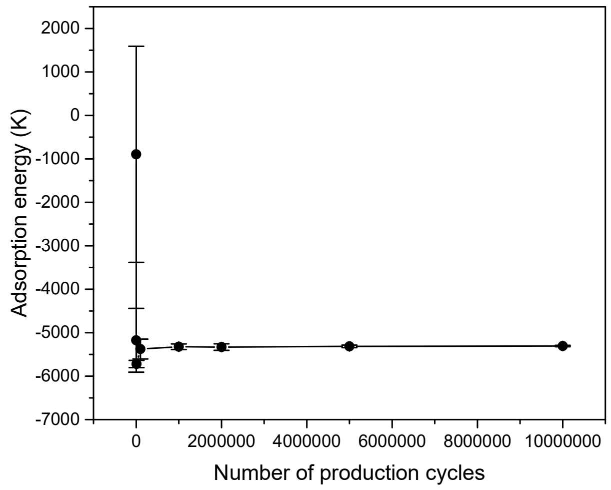

Figure S5: Evolution of adsorption energy accuracy according to the number of GCMC production cycles.

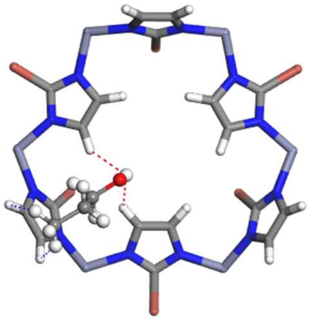

Figure S6: Interactions between propanol and ZIF-8_Br structure.

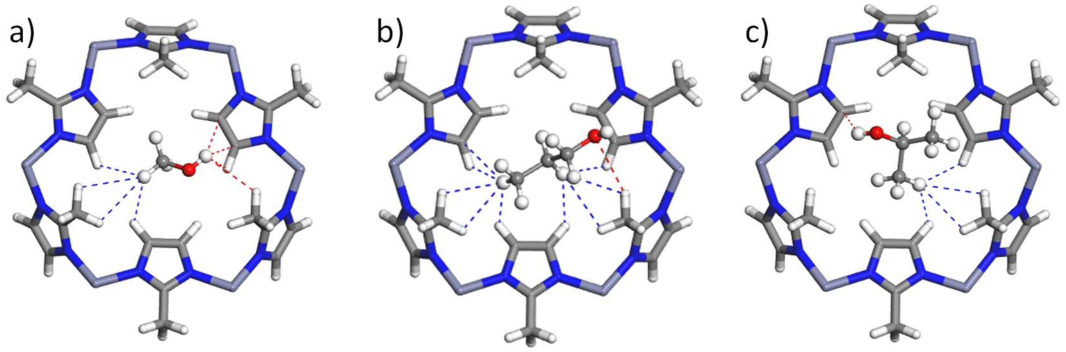

Figure S7: Host-guest interactions determined by periodic DFT for systems composed of ZIF-8 structure and a) methanol, b) n-propanol and c) isopropanol adsorbates.
